# Screening of Finnish *RAD51C* founder mutations in prostate and colorectal cancer patients

**DOI:** 10.1186/1471-2407-12-552

**Published:** 2012-11-23

**Authors:** Liisa M Pelttari, Riikka Nurminen, Alexandra Gylfe, Lauri A Aaltonen, Johanna Schleutker, Heli Nevanlinna

**Affiliations:** 1 Departments of Obstetrics and Gynecology, Helsinki University Central Hospital and University of Helsinki, Helsinki, Finland; 2 Institute of Biomedical Technology/BioMediTech, University of Tampere and Fimlab Laboratories, Tampere, Finland; 3 Department of Medical Genetics, Genome-Scale Biology Research Program, University of Helsinki, Helsinki, Finland; 4 Department of Medical Biochemistry and Genetics, University of Turku, Turku, Finland

**Keywords:** *RAD51C*, Prostate cancer, Colorectal cancer, Breast cancer, Ovarian cancer, Founder mutation

## Abstract

**Background:**

Rare, heterozygous germline mutations in the *RAD51C* gene have been found in breast and ovarian cancer families. In the Finnish population, we have identified two founder mutations in *RAD51C* that increase the risk of ovarian cancer but not breast cancer in the absence of ovarian cancer. Risk for other cancers has not been studied.

**Methods:**

To study the role of *RAD51C* mutations in other common cancer types, we genotyped the Finnish *RAD51C* founder mutations c.837 + 1G > A and c.93delG in 1083 prostate cancer patients and 802 colorectal cancer patients using TaqMan Real-Time PCR.

**Results:**

No *RAD51C* mutations c.837 + 1G > A or c.93delG were detected among the prostate or colorectal cancer patients.

**Conclusions:**

The results suggest that the *RAD51C* mutations do not predispose to prostate or colorectal cancer.

## Background

Mutations in cancer susceptibility genes may predispose the carriers to different cancer types. The most important high penetrance breast and ovarian cancer susceptibility genes are *BRCA1* and *BRCA2*[1] but, in addition to breast and ovarian cancer, germline mutations in these genes also increase the risk of prostate cancer [2,3] and in *BRCA2* also of pancreatic cancer [4]. In addition to the hereditary breast and ovarian cancer syndrome caused by germline mutations in *BRCA1* and *BRCA2*, ovarian cancer is also present in the Lynch syndrome that is caused by germline mutations in DNA mismatch repair genes and characterized by susceptibility to colorectal cancer [5]. Increased risk of breast cancer is also present in other rare cancer predisposition syndromes, namely Li-Fraumeni, Cowden and Peutz-Jeghers syndrome and ataxia-telangiectasia, which also predispose to other cancer types as well [1].

Biallelic mutations in *BRCA2* and in moderate penetrance breast cancer susceptibility genes *PALB2* and *BRIP1* cause Fanconi anemia, a chromosomal instability disorder characterized by developmental abnormalities, bone marrow failure, and cancer susceptibility, while heterozygous germline mutations increase the risk of breast cancer [6]. In addition to breast cancer, heterozygous mutations in *PALB2* also increase the risk of pancreatic cancer [4] but not prostate cancer [7,8]. In *RAD51C*, heterozygous germline mutations have been identified in breast and ovarian cancer families [9] and a homozygous missense mutation was found in a Fanconi anemia like disorder [10]. Following the original report, we and others have found rare deleterious mutations among breast and/or ovarian cancer families [11-17] but quite a few studies have not identified any clearly pathogenic mutations [18-24]. Indeed, the *RAD51C* mutations seem to be very rare. The association with ovarian cancer is well-established but whether the mutations also confer a risk for breast cancer remains controversial. Meindl *et al*. [9,25] and Osorio *et al*. [16] state that *RAD51C* mutations predispose to breast and ovarian cancer whereas Loveday *et al*. [17] estimated the relative risk of ovarian cancer for *RAD51C* protein truncating mutation carriers to be 5.88 but no elevated risk for breast cancer.

We recently sequenced the *RAD51C* gene in 277 Finnish breast and/or ovarian cancer families and identified two recurrent deleterious, protein truncating mutations c.837 + 1G > A and c.93delG and subsequently genotyped the mutations in a large series of breast and ovarian cancer patients and families [12]. The mutations significantly increased the risk of ovarian cancer but not breast cancer in the absence of ovarian cancer family history, indicating *RAD51C* as the first moderate penetrance susceptibility gene for ovarian cancer. The mutation carrier families shared a common haplotype indicating a founder effect for the mutations. Moreover, many of the mutation carriers had family history of various cancer types, including for instance colon and prostate cancer. Here we have genotyped the *RAD51C* mutations c.837 + 1G > A and c.93delG in 1083 prostate and 802 colorectal cancer patients to assess the risk for these cancer types. To our knowledge, the role of *RAD51C* mutations in other cancer types than breast and ovarian cancer has not been studied so far.

## Methods

### Subjects

The *RAD51C* mutations c.837 + 1G > A and c.93delG were genotyped in 1083 Finnish prostate cancer cases and 802 colorectal cancer cases. The prostate cancer patients included 894 unselected cases and 189 prostate cancer families. The unselected cases were collected from the Pirkanmaa Hospital District. The patients had been diagnosed with prostate cancer at the urology out-patient clinic at the Tampere University Hospital between years 1980 and 2008. All families included two or more affected members (the youngest affected male from each family was included in the analysis) and were collected as previously described [26]. The samples were genomic DNA isolated from peripheral blood with the Puregene kit, according to the manufacturer’s instructions (Gentra Systems, Inc., Minneapolis, MN, USA).

The colorectal cancer patients studied included 509 consecutive patients collected between May 1994 and April 1996 and 535 consecutive patients collected between March 1996 and June 1998 at nine large regional hospitals in southeastern Finland as described previously [27,28]. DNA was isolated from normal mucosa or blood by conventional DNA extraction methods. DNA was available from 802 patients. All the samples were screened for microsatellite instability (MSI) and the MSI positive cases were screened for *MLH1* and *MSH2* mutations. Of the 802 patients, 104 had family background of colorectal cancer and *MLH1* and *MSH2* mutations were identified in 19 cases.

This study was carried out according to the Helsinki declaration with informed consents from the patients and with permissions from Institutional Review Board of the Tampere University Hospital, The Ministry of Health and Social Affairs, National Authority for Medical Affairs, National Institute for Health and Welfare and Helsinki University Hospital Ethics Committee.

### Genotyping

We used TaqMan Custom Assays and TaqMan Genotyping MasterMix (Applied Biosystems, Foster City, CA, USA) to genotype the c.837 + 1G > A and c.93delG mutations. PCR was performed in 7500 Fast Real-Time PCR System or in 9800 Fast Thermal Cycler and genotype calling was performed with 7500 Fast Real-Time PCR System and ABI Prism 7500 SDS v1.4 software (Applied Biosystems, Foster City, CA, USA).

## Results and discussion

Most moderate penetrance mutations identified so far, including mutations in *RAD51C*, appear to be very rare with mostly unique mutations in out-bread populations. This poses considerable challenges for investigating them in large series of patients with different cancer types to determine association also with other cancers. We took here advantage of the *RAD51C* founder mutations in Finland and genotyped the c.837 + 1G > A and c.93delG mutations in 1083 prostate and 802 colorectal cancer patients, including also familial cases. No mutations were detected among the patients.

We previously detected the *RAD51C* c.837 + 1G > A or c.93delG mutations in 1.4% of Finnish ovarian cancer patients (in 1% of unselected ovarian cancer patients and in 25% of ovarian cancer families) compared to 0.2% in controls, indicating a significantly increased risk of ovarian cancer with also clinical implications for the carriers [12]. Moreover, the mutation carrier families were found to share a common haplotype indicating a shared ancestry for the families and a founder effect for the mutations in the Finnish population. Founder mutations are typical for isolated populations, such as the Finnish population, and they may explain a major fraction of all mutations in specific genes [29,30]. In the Finnish population, strong founder mutations have been detected for instance in the breast cancer susceptibility genes *BRCA1* and *BRCA2*[31] as well as in *PALB2*[32].

Some of the *RAD51C* mutation carriers in our previous study [12] had family history of diverse cancer types (abdomen, brain, bone marrow, cervical, colon, female genital, kidney, leukemia, liver, lung, melanoma, pancreas, prostate, skin, stomach, tongue, and ventricle) but unfortunately we did not have available samples for genotyping of these relatives. Few other studies have also identified *RAD51C* mutation carrier families with diverse cancer types [9,11,13,15-17]. This prompted us to study the effect of the mutations also on other common cancer types besides breast and ovarian cancer by screening of the founder mutations in an extensive series of prostate and colorectal cancer cases.

There is a clear genetic component in prostate cancer susceptibility and several common low-risk genomic loci for prostate cancer susceptibility have been found but no high-penetrance genes have been identified [33,34]. Linkage studies have suggested that a prostate cancer susceptibility gene may be located on chromosome 17q22 [35,36], a region including also the *RAD51C* locus, whereas genome-wide association studies (GWAS) have identified common low risk variants in 17q12 and 17q24 regions [37,38]. Finnish as well as other Northern European patients have been included both in the linkage and in the GWA studies [35,36,38]. Several low-risk loci have also been identified for colorectal cancer but 17q22 has not been implicated in colorectal cancer susceptibility [39]. It has been estimated that 15–30% of colorectal cancer may be hereditary and approximately one-quarter of the familial cases occur as part of inherited colorectal tumor syndromes, mainly the Lynch syndrome caused by mutations in DNA mismatch repair genes, such as *MSH2* and *MLH1*, and the familial adenomatous polyposis syndrome caused by mutations in the *APC* gene [40]. The absence of the *RAD51C* founder mutations among the prostate and colorectal cancer patients genotyped here suggests that *RAD51C* does not contribute to prostate or colorectal cancer predisposition. However, we cannot fully exclude a possible role of *RAD51C* as there might be mutation specific effects on cancer risk. To fully assess the role of *RAD51C* in susceptibility to other cancer types, screening of the whole coding region of the gene in large sample sets would be needed.

## Conclusions

In conclusion, we did not detect the Finnish founder mutations in the *RAD51C* gene among 1083 prostate and 802 colorectal cancer patients suggesting that the *RAD51C* mutations do not predispose to prostate or colorectal cancer. These results have clinical value in assessing the cancer risks associated with *RAD51C* mutations in the mutation carrier families.

## Competing interests

The authors declare that they have no competing interests.

## Authors’ contributions

LMP and HN designed the study and wrote the manuscript. LMP and RN carried out the genotyping. JS, AG and LA contributed samples and patient information. All authors read and approved the final manuscript.

## Pre-publication history

The pre-publication history for this paper can be accessed here:

http://www.biomedcentral.com/1471-2407/12/552/prepub

## References

[B1] Stratton MR Rahman N The emerging landscape of breast cancer susceptibility Nat Genet 2008 40 17 22 10.1038/ng.2007.53 18163131

[B2] Leongamornlert D Mahmud N Tymrakiewicz M Saunders E Dadaev T Castro E Goh C Govindasami K Guy M O’Brien L Sawyer E Hall A Wilkinson R Easton D Goldgar D Eeles R Kote-Jarai Z The UKGPCS Collaborators Germline BRCA1 mutations increase prostate cancer risk Br J Cancer 2012 106 1697 1701 10.1038/bjc.2012.146 22516946 PMC3349179

[B3] Kote-Jarai Z Leongamornlert D Saunders E Tymrakiewicz M Castro E Mahmud N Guy M Edwards S O’Brien L Sawyer E Hall A Wilkinson R Dadaev T Goh C Easton D Goldgar D Eeles R UKGPCS Collaborators BRCA2 is a moderate penetrance gene contributing to young-onset prostate cancer: implications for genetic testing in prostate cancer patients Br J Cancer 2011 105 1230 1234 10.1038/bjc.2011.383 21952622 PMC3208504

[B4] Klein AP Genetic susceptibility to pancreatic cancer Mol Carcinog 2012 51 14 24 10.1002/mc.20855 22162228 PMC3570154

[B5] Lynch HT Casey MJ Snyder CL Bewtra C Lynch JF Butts M Godwin AK Hereditary ovarian carcinoma: heterogeneity, molecular genetics, pathology, and management Mol Oncol 2009 3 97 137 10.1016/j.molonc.2009.02.004 19383374 PMC2778287

[B6] D’Andrea AD Susceptibility pathways in Fanconi’sanemia and breast cancer N Engl J Med 2010 362 1909 1919 10.1056/NEJMra0809889 20484397 PMC3069698

[B7] Tischkowitz M Sabbaghian N Ray AM Lange EM Foulkes WD Cooney KA Analysis of the gene coding for the BRCA2-interacting protein PALB2 in hereditary prostate cancer Prostate 2008 68 675 678 10.1002/pros.20729 18288683 PMC2683627

[B8] Pakkanen S Wahlfors T Siltanen S Patrikainen M Matikainen MP Tammela TL Schleutker J PALB2 Variants in hereditary and unselected Finnish prostate cancer cases J Negat Results Biomed 2009 8 12 10.1186/1477-5751-8-12 20003494 PMC2806404

[B9] Meindl A Hellebrand H Wiek C Erven V Wappenschmidt B Niederacher D Freund M Lichtner P Hartmann L Schaal H Ramser J Honisch E Kubisch C Wichmann HE Kast K Deissler H Engel C Muller-Myhsok B Neveling K Kiechle M Mathew CG Schindler D Schmutzler RK Hanenberg H Germline mutations in breast and ovarian cancer pedigrees establish RAD51C as a human cancer susceptibility gene Nat Genet 2010 42 410 414 10.1038/ng.569 20400964

[B10] Vaz F Hanenberg H Schuster B Barker K Wiek C Erven V Neveling K Endt D Kesterton I Autore F Fraternali F Freund M Hartmann L Grimwade D Roberts RG Schaal H Mohammed S Rahman N Schindler D Mathew CG Mutation of the RAD51C gene in a fanconianemia-like disorder Nat Genet 2010 42 406 409 10.1038/ng.570 20400963

[B11] Romero A Perez-Segura P Tosar A Garcia-Saenz JA Diaz-Rubio E Caldes T de la Hoya M A HRM-based screening method detects RAD51C germ-line deleterious mutations in Spanish breast and ovarian cancer families Breast Cancer Res Treat 2011 129 939 946 10.1007/s10549-011-1543-x 21537932

[B12] Pelttari LM Heikkinen T Thompson D Kallioniemi A Schleutker J Holli K Blomqvist C Aittomaki K Butzow R Nevanlinna H RAD51C is a susceptibility gene for ovarian cancer Hum Mol Genet 2011 20 3278 3288 10.1093/hmg/ddr229 21616938

[B13] Vuorela M Pylkas K Hartikainen JM Sundfeldt K Lindblom A von WachenfeldtWappling A Haanpaa M Puistola U Rosengren A Anttila M Kosma VM Mannermaa A Winqvist R Further evidence for the contribution of the RAD51C gene in hereditary breast and ovarian cancer susceptibility Breast Cancer Res Treat 2011 130 1003 1010 10.1007/s10549-011-1677-x 21750962

[B14] Walsh T Casadei S Lee MK Pennil CC Nord AS Thornton AM Roeb W Agnew KJ Stray SM Wickramanayake A Norquist B Pennington KP Garcia RL King MC Swisher EM Mutations in 12 genes for inherited ovarian, fallopian tube, and peritoneal carcinoma identified by massively parallel sequencing Proc Natl Acad Sci U S A 2011 108 18032 18037 10.1073/pnas.1115052108 22006311 PMC3207658

[B15] Thompson ER Boyle SE Johnson J Ryland GL Sawyer S Choong DY Chenevix-Trench G Trainer AH Lindeman GJ Mitchell G James PA Campbell IG kConFab Analysis of RAD51C germline mutations in high-risk breast and ovarian cancer families and ovarian cancer patients Hum Mutat 2012 33 95 99 10.1002/humu.21625 21990120

[B16] Osorio A Endt D Fernandez F Eirich K Hoya MD Schmutzler R Caldes T Meindl A Schindler D Benitez J Predominance of pathogenic missense variants in the RAD51C gene occurring in breast and ovarian cancer families Hum Mol Genet 2012 21 2889 2898 10.1093/hmg/dds115 22451500

[B17] Loveday C Turnbull C Ruark E Xicola RM Ramsay E Hughes D Warren-Perry M Snape K Eccles D Evans DG Gore M Renwick A Seal S Antoniou AC Rahman N Breast Cancer Susceptibility Collaboration (BCSC) (UK) Germline RAD51C mutations confer susceptibility to ovarian cancer Nat Genet 2012 44 475 476 10.1038/ng.2224 22538716

[B18] Zheng Y Zhang J Hope K Niu Q Huo D Olopade OI Screening RAD51C nucleotide alterations in patients with a family history of breast and ovarian cancer Breast Cancer Res Treat 2010 124 857 861 10.1007/s10549-010-1095-5 20697805

[B19] Akbari MR Tonin P Foulkes WD Ghadirian P Tischkowitz M Narod SA RAD51C germline mutations in breast and ovarian cancer patients Breast Cancer Res 2010 12 404 10.1186/bcr2619 20723205 PMC2949649

[B20] Wong MW Nordfors C Mossman D Pecenpetelovska G Avery-Kiejda KA Talseth-Palmer B Bowden NA Scott RJ BRIP1, PALB2, and RAD51C mutation analysis reveals their relative importance as genetic susceptibility factors for breast cancer Breast Cancer Res Treat 2011 127 853 859 10.1007/s10549-011-1443-0 21409391

[B21] Pang Z Yao L Zhang J Ouyang T Li J Wang T Fan Z Fan T Lin B Xie Y RAD51C germline mutations in Chinese women with familial breast cancer Breast Cancer Res Treat 2011 129 1019 1020 10.1007/s10549-011-1574-3 21597919

[B22] Clague J Wilhoite G Adamson A Bailis A Weitzel JN Neuhausen SL RAD51C germline mutations in breast and ovarian cancer cases from high-risk families PLoS One 2011 6 9 e25632 10.1371/journal.pone.0025632 21980511 PMC3182241

[B23] De Leeneer K Van Bockstal M De Brouwer S Swietek N Schietecatte P Sabbaghian N Van den Ende J Willocx S Storm K Blaumeiser B Van Asperen CJ Wijnen JT Leunen K Legius E Michils G Matthijs G Blok MJ Gomez-Garcia E De Paepe A Tischkowitz M Poppe B Claes K Evaluation of RAD51C as cancer susceptibility gene in a large breast-ovarian cancer patient population referred for genetic testing Breast Cancer Res Treat 2012 133 393 398 10.1007/s10549-012-1998-4 22370629

[B24] Lu W Wang X Lin H Lindor NM Couch FJ Mutation screening of RAD51C in high-risk breast and ovarian cancer families Fam Cancer 2012 11 381 385 10.1007/s10689-012-9523-9 22476429 PMC3418444

[B25] Meindl A Eirich K Engert S Becker A Endt D Ditsch N Schmutzler RK Schindler D Germline RAD51C mutations confer susceptibility to ovarian cancer Nat Genet 2012 44 476 10.1038/ng.2223 22538716

[B26] Matikainen MP Sankila R Schleutker J Kallioniemi OP Pukkala E Nationwide cancer family ascertainment using Finnish cancer registry data on family names and places of birth for 35,761 prostate cancer patients Int J Cancer 2000 88 307 312 10.1002/1097-0215(20001015)88:2<307::AID-IJC25>3.0.CO;2-7 11004685

[B27] Aaltonen LA Salovaara R Kristo P Canzian F Hemminki A Peltomaki P Chadwick RB Kaariainen H Eskelinen M Jarvinen H Mecklin JP de la Chapelle A Incidence of hereditary nonpolyposis colorectal cancer and the feasibility of molecular screening for the disease N Engl J Med 1998 338 1481 1487 10.1056/NEJM199805213382101 9593786

[B28] Salovaara R Loukola A Kristo P Kaariainen H Ahtola H Eskelinen M Harkonen N Julkunen R Kangas E Ojala S Tulikoura J Valkamo E Jarvinen H Mecklin JP Aaltonen LA de la Chapelle A Population-based molecular detection of hereditary nonpolyposis colorectal cancer J ClinOncol 2000 18 2193 2200 10.1200/JCO.2000.18.11.2193 10829038

[B29] Arcos-Burgos M Muenke M Genetics of population isolates Clin Genet 2002 61 233 247 10.1034/j.1399-0004.2002.610401.x 12030885

[B30] Peltonen L Jalanko A Varilo T Molecular genetics of the Finnish disease heritage Hum Mol Genet 1999 8 1913 1923 10.1093/hmg/8.10.1913 10469845

[B31] Sarantaus L Huusko P Eerola H Launonen V Vehmanen P Rapakko K Gillanders E Syrjakoski K Kainu T Vahteristo P Krahe R Paakkonen K Hartikainen J Blomqvist C Lopponen T Holli K Ryynanen M Butzow R Borg A WastesonArver B Holmberg E Mannermaa A Kere J Kallioniemi OP Winqvist R Nevanlinna H Multiple founder effects and geographical clustering of BRCA1 and BRCA2 families in Finland Eur J Hum Genet 2000 8 757 763 10.1038/sj.ejhg.5200529 11039575

[B32] Erkko H Xia B Nikkila J Schleutker J Syrjakoski K Mannermaa A Kallioniemi A Pylkas K Karppinen SM Rapakko K Miron A Sheng Q Li G Mattila H Bell DW Haber DA Grip M Reiman M Jukkola-Vuorinen A Mustonen A Kere J Aaltonen LA Kosma VM Kataja V Soini Y Drapkin RI Livingston DM Winqvist R A recurrent mutation in PALB2 in Finnish cancer families Nature 2007 446 316 319 10.1038/nature05609 17287723

[B33] Schaid DJ The complex genetic epidemiology of prostate cancer Hum Mol Genet 2004 13 Suppl 1 R103 R121 14749351 10.1093/hmg/ddh072

[B34] Kim ST Cheng Y Hsu FC Jin T Kader AK Zheng SL Isaacs WB Xu J Sun J Prostate cancer risk-associated variants reported from genome-wide association studies: meta-analysis and their contribution to genetic variation Prostate 2010 70 1729 1738 20564319 10.1002/pros.21208 PMC3013361

[B35] Gillanders EM Xu J Chang BL Lange EM Wiklund F Bailey-Wilson JE Baffoe-Bonnie A Jones M Gildea D Riedesel E Albertus J Isaacs SD Wiley KE Mohai CE Matikainen MP Tammela TL Zheng SL Brown WM Rokman A Carpten JD Meyers DA Walsh PC Schleutker J Gronberg H Cooney KA Isaacs WB Trent JM Combined genome-wide scan for prostate cancer susceptibility genes J Natl Cancer Inst 2004 96 1240 1247 10.1093/jnci/djh228 15316059

[B36] Cropp CD Simpson CL Wahlfors T Ha N George A Jones MS Harper U Ponciano-Jackson D Green TA Tammela TL Bailey-Wilson J Schleutker J Genome-wide linkage scan for prostate cancer susceptibility in Finland: evidence for a novel locus on 2q37.3 and confirmation of signal on 17q21-q22 Int J Cancer 2011 129 10 2400 2407 10.1002/ijc.25906 21207418 PMC3137914

[B37] Gudmundsson J Sulem P Steinthorsdottir V Bergthorsson JT Thorleifsson G Manolescu A Rafnar T Gudbjartsson D Agnarsson BA Baker A Sigurdsson A Benediktsdottir KR Jakobsdottir M Blondal T Stacey SN Helgason A Gunnarsdottir S Olafsdottir A Kristinsson KT Birgisdottir B Ghosh S Thorlacius S Magnusdottir D Stefansdottir G Kristjansson K Bagger Y Wilensky RL Reilly MP Morris AD Kimber CH Two variants on chromosome 17 confer prostate cancer risk, and the one in TCF2 protects against type 2 diabetes Nat Genet 2007 39 977 983 10.1038/ng2062 17603485

[B38] Jin G Lu L Cooney KA Ray AM Zuhlke KA Lange EM Cannon-Albright LA Camp NJ Teerlink CC Fitzgerald LM Stanford JL Wiley KE Isaacs SD Walsh PC Foulkes WD Giles GG Hopper JL Severi G Eeles R Easton D Kote-Jarai Z Guy M Rinckleb A Maier C Vogel W Cancel-Tassin G Egrot C Cussenot O Thibodeau SN McDonnell SK Validation of prostate cancer risk-related loci identified from genome-wide association studies using family-based association analysis: evidence from the international consortium for prostate cancer genetics (ICPCG) Hum Genet 2012 131 1095 1103 10.1007/s00439-011-1136-0 22198737 PMC3535428

[B39] Varghese JS Easton DF Genome-wide association studies in common cancers–what have we learnt? Curr Opin Genet Dev 2010 20 3 201 209 10.1016/j.gde.2010.03.012 20418093

[B40] Fearon ER Molecular genetics of colorectal cancer Annu Rev Pathol 2011 6 479 507 10.1146/annurev-pathol-011110-130235 21090969

